# Sleep Neuroimaging and Models of Consciousness

**DOI:** 10.3389/fpsyg.2013.00256

**Published:** 2013-05-13

**Authors:** Enzo Tagliazucchi, Marion Behrens, Helmut Laufs

**Affiliations:** ^1^Neurology Department and Brain Imaging Center, Goethe University FrankfurtFrankfurt am Main, Germany; ^2^Department of Neurology, Schleswig Holstein University HospitalKiel, Germany

**Keywords:** consciousness, sleep, fMRI, EEG, resting state

## Abstract

Human deep sleep is characterized by reduced sensory activity, responsiveness to stimuli, and conscious awareness. Given its ubiquity and reversible nature, it represents an attractive paradigm to study the neural changes which accompany the loss of consciousness in humans. In particular, the deepest stages of sleep can serve as an empirical test for the predictions of theoretical models relating the phenomenology of consciousness with underlying neural activity. A relatively recent shift of attention from the analysis of evoked responses toward spontaneous (or “resting state”) activity has taken place in the neuroimaging community, together with the development of tools suitable to study distributed functional interactions. In this review we focus on recent functional Magnetic Resonance Imaging (fMRI) studies of spontaneous activity during sleep and their relationship with theoretical models for human consciousness generation, considering the global workspace theory, the information integration theory, and the dynamical core hypothesis. We discuss the venues of research opened by these results, emphasizing the need to extend the analytic methodology in order to obtain a dynamical picture of how functional interactions change over time and how their evolution is modulated during different conscious states. Finally, we discuss the need to experimentally establish absent or reduced conscious content, even when studying the deepest sleep stages.

## 1 Introduction

Non-Rapid Eye Movement (NREM) sleep in humans is commonly defined as a state characterized by suspended sensory activity, unresponsiveness to stimuli, a relative muscular inactivity, and loss of conscious awareness (AASM, 2007; Tononi, 2009). A well equipped researcher could unequivocally establish the first three features, since they represent objective qualities whose presence can be assessed by suitable measurements. The fourth feature, in contrast, is of a more puzzling nature. The presence or absence of conscious awareness appears difficult to establish from a third person perspective, given its subjective and private nature. Furthermore, the loss of consciousness is dissociated from the first three features, as philosophers have speculated by introducing the concept of “zombie agents” (Chalmers, 1996) and as happens, for instance, during complex partial seizures (Blumenfeld and Taylor, 2003). The particular nature of this loss of conscious awareness has even led to question the suitability of traditional scientific methodology to explain this phenomenon (McGinn, 1989), which consists of a quintessentially subjective experience.

It is certainly unknown whether neuroscience and the study of the brain will ever solve the so-called “hard problem,” i.e., to explain the subjective characteristics acquired by conscious percepts or, in other words, why do we feel things and why they feel the way they do (Chalmers, 1995). However, it is likely that the neural mechanisms underlying consciousness can be discovered, even if in the end they do not tell us anything about the subjective side of perception. In this picture, consciousness is a product of certain neural processes which must account for its phenomenology (knowledge about the conscious state which is obtained by experimentation or introspection). Examples of this knowledge include the serial nature of conscious operations, the possibility of subliminal perception, the attentional blink, the unified nature of each conscious scene, and many others (for a more comprehensive discussion, see Edelman, 2003; Seth et al., 2006).

While a discovery science of consciousness is possible, investigations are greatly aided by theoretical models of how brain neurophysiology can give rise to these phenomenological features (Seth, 2007). Such a model needs to postulate a causal link between neural observables (such as firing rate or synchrony, spiking frequency, activity in different cortical structures, etc.) and these features. As a simple (and ultimately wrong) example, it could be postulated that loss of consciousness during sleep is caused by a suspension of neural activity, i.e., firing rates drop, impairing the cortical information processing which gives rise to conscious content. This model appears plausible, since cortical activity is linked (for example, by lesion studies) to the contents of consciousness and firing rate modulation is considered a fundamental mechanism of neural information processing (Gerstner et al., 1997). We know that neural firing rates can be maintained or even increased during sleep (Tononi and Massimini, 2008) and therefore this model is not correct, but it should nevertheless illustrate the procedure according to which such models can be formulated.

Here we will review more successful models of consciousness, including the global workspace model, the information integration theory, and the dynamical core hypothesis. These choices are motivated by the relatively large number of neuroimaging studies which address their predictions. More comprehensive reviews can be found elsewhere (Seth, 2007; Boly and Seth, 2012), here we wish to focus on those models best suited to be approached by the study of spontaneous (or “resting state”) activity recorded with functional Magnetic Resonance Imaging (fMRI). At first glance, this choice appears rather arbitrary and driven by the experimental methodology. However, a common characteristic of these models is their treatment of consciousness as a global, dynamic process (instead of as a localized phenomenon). This is consistent with the experimental observation that consciousness can survive localized cortical damage and will fade only if sub-cortical arousal systems are compromised (Tononi and Edelman, 1998). Since fMRI is currently the only neuroimaging technique allowing to non-invasively record whole-brain activity with spatial resolution of millimeters at a reasonable temporal resolution (≈1 s), it provides a promising experimental set-up to address predictions about global brain function and dynamics.

A natural starting point to test a theoretical model linking neural activity to conscious phenomenology is to contrast conscious vs. unconscious brain states. From an experimental viewpoint, NREM sleep is attractive since it is recurrent, evolves deterministically through a series of sleep stages until a state of deep unconsciousness is reached and is reversible (every subject can be studied both in a conscious and unconscious state). Disorders of consciousness such as a coma or the vegetative state usually involve brain trauma or injuries, on the other hand, sleep is a physiological state occurring in the healthy brain. Motivated by this, we will focus on human sleep and how sleep neuroimaging experiments can be used to test the predictions of the aforementioned theories. After reviewing the main published results, we will discuss future venues of research, such as the need to fully incorporate a temporal dimension and to track the dynamical changes of brain connectivity over time. Finally, we will discuss to what extent deep NREM sleep can be regarded as a truly unconscious state and what possible measures can be taken to control for this in the experimental paradigm.

## 2 The Global Workspace Theory and Frontoparietal Connectivity

The global workspace theory (introduced by Baars, 1988) postulates that consciousness serves an integrative role. Conscious content is characterized by its global availability and can be accessed by different cognitive processes, i.e., it is broadcasted to an “audience” of distributed, parallel networks which can process information unconsciously. The integration of information into a global resource naturally accounts for the serial nature of conscious experience. A neural instantiation of the global workspace theory has been proposed by Dehaene et al. (2003), in which stimulation leads to the generation of a coherent and global activity pattern inhibiting the processing of concurrent stimuli. As stimuli salience is increased, a sudden (i.e., non-linear) transition from a local to a global activity pattern takes place.

A direct prediction of the global workspace theory is that unconscious stimuli will activate cortical areas restricted to low level sensory processing, whereas the passage to consciousness will involve activity in a constellation of regions transcending the sensory cortices. Experimental results assign to a frontoparietal network of regions a key role in this global pattern of activity. This fact is supported by experiments contrasting conscious vs. unconscious perception (e.g., Dehaene et al., 2001) and by the increased metabolism of these regions during resting state wakefulness (Mazoyer et al., 2001; Raichle, 2006). Furthermore, resting state activity Blood Oxygen Level Dependent (BOLD) activity fluctuations are coherent in a frontoparietal network of regions which has been termed the Default Mode Network (DMN), since activity in these areas decreases during attention demanding tasks and has a positive association with introspection and self-awareness (Raichle et al., 2001).

Auditory stimulation during deep sleep results in a local activation of auditory cortices (Portas et al., 2000), which is concordant with the predictions of the global workspace theory. It must be noted, however, that studies of auditory stimulation during sleep assume a BOLD response linearly correlated with the model. Recent results show a global brain response in event-related paradigms, with activity changes departing from a simple covariance with the time series of expected BOLD activation (Gonzalez-Castillo et al., 2012). This point should be taken into consideration for future studies studying the neural responses elicited by stimulation during unconscious states, such as sleep.

**Figure 1:**
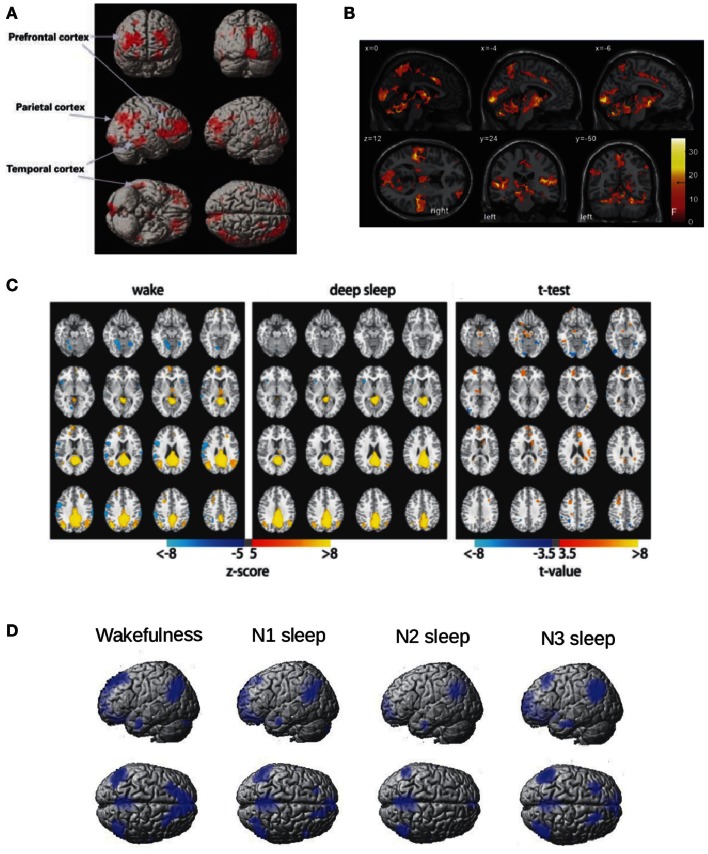
**Frontoparietal metabolism and connectivity during NREM sleep**. **(A)** Regions of decreased glucose metabolism during NREM sleep compared to wakefulness adapted from Nofzinger et al. (2002). Figure reproduced with permission from Oxford University Press. **(B)** Regions showing a positive BOLD response to K-complexes during the N2 stage of NREM sleep adapted from Jahnke et al. (2012) thresholded at *F* > 18.7, *p* < 0.05, FWE corrected. Figure reproduced with permission from Elsevier. **(C)** Seed correlation maps based on PCC during wakefulness and deep sleep (*z* > 5) and for their difference (*z* > 3.5) adapted from Horovitz et al. (2009). **(D)** The Default Mode Network (DMN) obtained with Independent Component Analysis (ICA) for wakefulness, N1, N2, and N3 sleep (the fMRI data used was previously reported in Tagliazucchi et al., 2013).

During sleep, not only auditory stimulation fails to elicit activity in the frontoparietal network, but in addition decreased metabolism is observed in these regions (Nofzinger et al., 2002, see Figure 1A for an illustration). Recent EEG-fMRI studies have extensively studied resting state functional connectivity of BOLD signals inside the frontoparietal DMN, finding decreased connectivity between the frontal and posterior components (Horovitz et al., 2009; Sämann et al., 2011) (Figure 1C). Changes in connectivity in other brain systems (such as sensory cortices) were not found during light sleep (Larson-Prior et al., 2009) and have not been intensively evaluated for deeper sleep stages (with the exception of a control network anticorrelated with the DMN, studied in Sämann et al., 2011). Thus, it has not been firmly established whether sleep correlates with a global connectivity breakdown or only with a frontoparietal disconnection. However, the study of electrophysiological events (EEG microstates) correlated with different networks measured with fMRI (Britz et al., 2010) shows invariant results (in terms of preserved topographic maps) across NREM sleep (Brodbeck et al., 2012).

Further evidence of the link between DMN activity and conscious wakefulness can be obtained by studying the BOLD correlates of K-complexes, large amplitude EEG events which are hypothesized to serve a dual function: protection against environmental stimuli (Bastien et al., 2000; Cash et al., 2009) and brief arousals to monitor the surroundings (Ehrhart et al., 1981; Halasz, 1993). Combined EEG-fMRI studies show that the BOLD correlates of K-complexes comprise multimodal sensory areas as well as key regions in the DMN (e.g., the precuneus) (Caporro et al., 2012; Jahnke et al., 2012) (the resulting activation patterns are shown in Figure 1B), which is consistent with the latter proposed function (brief periods of a “wakefulness-like” brain state).

An interesting observation is that deep sleep disrupts the coherence of spontaneous BOLD fluctuations inside the DMN, but does not completely eliminate the network. For instance, a model free approach to identify coherent activity fluctuations (Independent Component Analysis – ICA; Beckmann et al., 2005) identifies the DMN both during wakefulness and deep sleep. The resulting DMN ICA components (obtained using the data reported in Tagliazucchi et al., 2013) are shown in Figure 1D (but see also Boly et al., 2012). If the emergence of this network is related to a conscious state, how can it be generally preserved during sleep? An answer to this question can be found in the non-trivial large-scale thalamocortical connectivity which correlates with BOLD functional connectivity (Honey et al., 2007; Greicius et al., 2009; Hermundstad et al., 2013). Homeostatic processes could result in preserved connectivity even during deep sleep-setting up a baseline state of the brain including a functional repertoire resembling different task-related states and thus facilitating a quick response to environmental demands (Raichle, 2010). If this indeed occurs during unconscious states such as sleep, it follows that measures of functional connectivity must be “normalized” by the strength of the underlying anatomical connections in order to properly understand changes in frontoparietal connectivity.

## 3 Information Integration

Two remarkable phenomenological characteristics of every conscious scene are its highly integrated, indivisible nature, and its high information content. These two observations have been developed by Tononi (2004, 2008) into a mathematically grounded theory of consciousness. As will be briefly explained below, the combination of these two features requires information integration, which means that the system as a whole generates more information than what would be expected from the independent contribution of all its parts.

Information here is understood in terms of uncertainty reduction, i.e., it quantifies how much knowledge about the system is gained when a particular state (out of many possible states) is specified. For instance, knowing the state of a unit with two possible states (such as a photodiode, a recurrent example) will yield only one bit of information. The repertoire of conscious states is immensely larger and therefore each of its states entails a much higher informational content. Such a high informational content could also be achieved, in principle, by assembling a large number of binary units together, which would exponentially increase the number of possible states of the system. However, this information is not integrated, since the units do not interact and therefore the informational content of the system as a whole does not go beyond the sum of each independent contribution. Furthermore, there is no casual dependence between the units of such system, since changes induced in any subset of units will not affect the remaining ones.

This model argues that conscious awareness is a function of a system’s capacity for information integration. From a theoretical point of view, this is attractive since the information integration capacity of any physical system can be quantified in a single numerical quantity, termed Φ (Tononi and Sporns, 2003). Furthermore, the system subsets in which information is integrated-termed complexes can be algorithmically identified. To measure Φ, a system is first divided into two non-overlapping subsets *A* and *B*. Then, the states of one partition are replaced by random noise (i.e., the maximum entropy distribution). The effective information is then obtained as the relative entropy of *B* with and without the noise injection. This procedure is repeated inverting the order of the subsets and the effective informations are added. Finally, the weakest informational link is identified as the Minimum Information Partition (MIP), this is, the division of the system yielding the minimum amount of effective information. This value, properly normalized, is Φ. The information integrating subsets, i.e., the complexes, are identified as those sets which have no sub- or super-sets with higher Φ value (or equivalently, none of their subsets integrates more information than them and they are not part of a larger set integrating more information than them).

Following the predictions of this model, states of diminished conscious awareness – such as deep NREM sleep should be characterized by impaired information integration, i.e., a lower Φ. Simulations suggest that information integration is decreased for systems whose units behave in a synchronous way, since the repertoire of possible states is reduced (Balduzzi and Tononi, 2008). During deep human NREM sleep cortical dynamics become bistable, with periods of firing (depolarized up-state) alternating with periods of quiescence (hyperpolarized down-state). These dynamics are manifest in the EEG as large amplitude waves whose frequency (in the <4 Hz range) is much lower than that of rhythms observed during wakefulness (interestingly, BOLD activity fluctuations also increase in amplitude during sleep Horovitz et al., 2008, but it is unknown whether these two effects are related). Computer simulations also suggest that high values of information integration require network architectures displaying both functional specialization (i.e., segregated functional processes) and functional integration between them (Tononi and Sporns, 2003).

**Figure 2:**
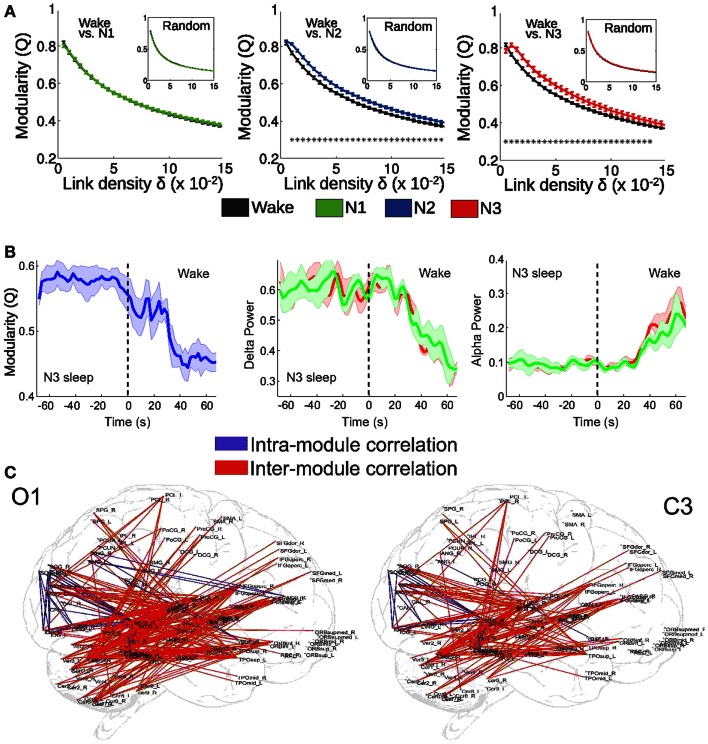
**fMRI functional connectivity networks show a more segregated structure during deep NREM sleep**. **(A)** Functional network modularity (Q) of wakefulness vs. N1, N2, and N3 sleep (*p < 0.05). Results are plotted against the network density δ (a function of the correlation threshold, a free parameter for network construction). **(B)** Modularity, delta, and alpha EEG power during arousals (transitions from sleep to wakefulness). Two different EEG channels were used to extract EEG alpha and delta power: occipital O1 (red) and central C3 (green). **(C)** Anatomical overlay of links representing negative correlations between functional connectivity fluctuations of the connected regions and spontaneous fluctuations of EEG delta power (thresholded at *p* < 0.05, Bonferroni corrected). Adapted from Tagliazucchi et al. (2013). Figure reproduced with permission from Elsevier.

By means of EEG-fMRI recordings during wakefulness and sleep, we recently tested these two predictions (Tagliazucchi et al., 2013). More specifically, we hypothesized that deep sleep would: (1) result in an alteration of the integration-segregation balance of brain activity and (2) that this alteration would correlate with the power of slow cortical rhythms (concurrently recorded with scalp EEG). We represented whole-brain interactions as a functional network, in which each node corresponds to a cortical or sub-cortical area and connections between them indicate synchronous activity of their BOLD signals (Sporns et al., 2004; Bullmore and Sporns, 2009). The degree of functional integration/segregation in this structure can be quantified by the modularity (Q), which takes values between 0 and 1 and indicates how well the network can be separated into densely connected subsets (modules) sparsely connected between them (Sporns, 2013). As shown in Figure 2A, functional network modularity was found to increase during the deeper sleep stages (N2 and N3 sleep, following the AASM sleep staging criteria), which are characterized by impaired or absent consciousness. On the other hand, modularity was not found to increase during early (N1) sleep. Furthermore, modularity was found to correlate with the power of slow EEG rhythms, both during arousals from deep sleep to wakefulness (Figure 2B) and spontaneously (Figure 2C). These results are along the lines of the predicted integration/segregation disruption taking place in deep NREM sleep. However, the network modularity of a system is not necessarily equivalent to its Φ, which measures effective information and causal interactions. Since changes in correlation do not imply changes in causality (and vice-versa), it follows that any approach which is based on functional network properties can only yield an indirect confirmation of these predictions. A perturbational approach based on non-invasive magnetic stimulation shows that effective connectivity (measured using EEG) is impaired during NREM sleep (Massimini et al., 2005), but a similar approach has not been attempted so far during fMRI recordings. On the other hand, there are computational similarities between modularity and measures of neural complexity (Tononi et al., 1994), also introduced as potential correlates of the level of conscious awareness (see section on the dynamical core hypothesis). The finding of increased functional modularity during NREM sleep has been independently reported by other research groups (Boly et al., 2012; Spoormaker et al., 2012). Also, other network attributes associated with integration and segregation are altered during human NREM sleep (Spoormaker et al., 2010; Uehara et al., 2013), in fact allowing sleep classification solely based on fMRI functional connectivity information (Tagliazucchi et al., 2012c).

Φ is a global property of the thalamocortical system, and since fMRI can simultaneously record activity in the whole-brain with relatively good (when compared to other non-invasive methods) spatial resolution, it appears that the theoretical machinery proposed by Tononi and others could be directly applied on fMRI data to precisely quantify the thalamocortical Φ, as well as the complexes in which information is integrated. However, there are practical and theoretical obstacles to this program. First, the computation of Φ requires the identification of the MIP, which can only be done by exhaustively evaluating the effective information across all pairs of non-overlapping subsets. In practice, this computation is impossible for the number of voxels recorded in an fMRI experiment. Theoretically, Φ can take different values for a same system when considered at different spatial and temporal scales, and hence the scales at which the system optimally integrates information need to be first identified (for a clear discussion, see Tononi, 2010). fMRI records a metabolic signal correlated with synaptic processing in gray matter regions of ≈1 mm^3^ with temporal resolution in the range of 1 s. For computational reasons, it is a common procedure to reduce the dimensionality using anatomical information or clustering procedures (such as ICA). It is certainly not clear whether this is the adequate spatio-temporal scale. Also, dimensionality reduction can only be justified if units are aggregated into a complex (Tononi, 2010), but this assumption is difficult to justify without first identifying all complexes in the system.

## 4 The Dynamical Core Hypothesis

Experimental observations strongly suggest that the thalamocortical system is crucial for consciousness. Not only regions outside this system are irrelevant in terms of conscious content, but also localized thalamocortical lesions can be compatible with preserved consciousness (Tononi and Edelman, 1998). Empirical evidence further suggests that neural firing in a distributed subset of thalamocortical neurons occurs in response to any given conscious percept. It has been suggested by Tononi and Edelman (Tononi and Edelman, 1998; Edelman and Tononi, 2000) that such a group of neurons must have the following properties: (1) It must be a functional cluster, i.e., consist of neurons with stronger interactions between them than with the rest of the thalamocortical system (a concept analogous to that of the functional modules introduced in the previous section), (2) This functional cluster must be highly differentiated, i.e., must admit a large repertoire of possible activity patterns. These two properties follow from the two main phenomenological features of consciousness discussed in the previous section (its unified, undecomposable nature, and its high informability).

At any given time, in order to contribute to conscious content a neuron or group of neurons must participate in this thalamocortical functional cluster, termed “dynamical core.” This name emphasizes the changing nature of the functional cluster: its configuration is postulated to constantly change (at a temporal scale of hundreds of milliseconds) to accommodate the ongoing stream of conscious perception and mentation. Therefore, it is a categorical error to discuss where in the brain consciousness takes place or which neurons are engaged in its generation, since consciousness is a dynamical process which constantly reorganizes and always comprises a highly differentiated thalamocortical functional cluster.

**Figure 3:**
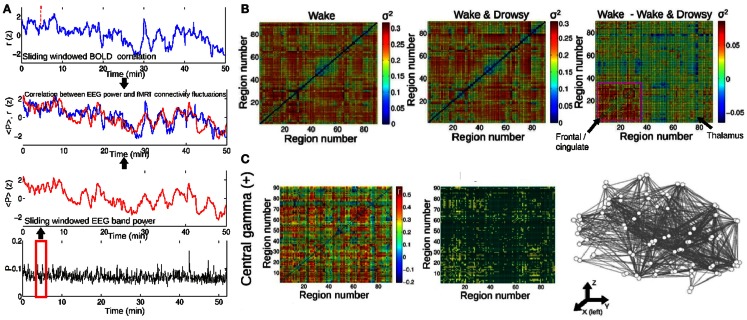
**The temporal evolution of BOLD synchronization is correlated with the power of EEG oscillations in different frequency bands**. **(A)** Procedure followed to study the relationship between fMRI functional connectivity changes and EEG power. A sliding window procedure is applied to obtain the time-dependent Pearson correlation between all pairs of BOLD signals extracted from any given brain parcellation. The same sliding window is then applied to obtain the evolution of EEG power in different frequency bands. **(B)** The temporal variance (σ^2^) of functional connectivity fluctuations between all pairs of regions. Results are for awake subjects and for subjects undergoing transitions to light sleep (drowsiness). On the right, the difference between both conditions is plotted. **(C)** Pairs of regions showing a positive correlation between BOLD functional connectivity and central EEG power in the gamma band (30–60 Hz). Figure adapted from Tagliazucchi et al. (2012b).

On a different temporal scale, it has been recently discovered that BOLD functional connectivity is dynamical and widely fluctuates over time (Chang and Glover, 2010; Tagliazucchi et al., 2011, 2012a; Handwerker et al., 2012; Hutchison et al., 2012; Smith et al., 2012). These fluctuations occur in the range between seconds and minutes, in contrast to the functional connectivity changes in the hundreds of milliseconds which are involved in the formation of the dynamical core. This different scale does not eliminate the possibility that dynamical BOLD connectivity is a manifestation of accumulated changes taking place in a faster temporal range. Furthermore, we have recently shown that these sudden BOLD functional connectivity changes can be traced to fluctuations in frequency-specific local neural synchronization, as indexed by scalp EEG power (Tagliazucchi et al., 2012b) (similar results were also independently obtained by another research group; Chang et al., 2013) and that they are predicted by conceptual models of brain activity with realistic anatomical connectivity (Haimovici et al., 2013). Insights on the dynamical nature of conscious processes could be then obtained by analyzing how BOLD dynamical functional connectivity is modulated across different conscious states. The dynamical core hypothesis predicts that, while average connectivity can remain the same (or be even higher) during sleep, its temporal variance will decrease when compared to that of wakefulness. This result was confirmed for frontal connectivity in subjects undergoing transitions to light sleep (Tagliazucchi et al., 2012b), following the method outlined in Figure 3A. The resulting variances are shown in Figure 3B for wakefulness, for subjects undergoing transitions to light sleep and for the difference. It can be expected that capitalizing on new tools to map dynamical changes of network modular structure (Bassett et al., 2011), this line of research could also lead to the identification of the most stable regions within different dynamical functional clusters.

Of particular interest here is the relationship between BOLD connectivity and EEG power in the relatively fast gamma (30–60 Hz) band (Tagliazucchi et al., 2012b), since it has been demonstrated that synchronization in this frequency range subserves conscious perception (Melloni et al., 2007). Tellingly, correlations between BOLD synchronization and gamma power were not found for subjects undergoing transitions to early sleep. Furthermore, synchronization in the gamma range has been proposed as a solution to the binding problem and could be of fundamental importance for the formation of the dynamical core (Treisman, 1996). Taken together, these results show that large-scale dynamical synchronization between a widespread network of brain regions can be predicted from gamma power and can be modulated by vigilance fluctuations, putting in evidence the importance of dynamical functional connectivity for the maintenance of wakeful rest.

## 5 Consciousness during NREM Sleep

The previous discussions rely on the everyday experience of deep sleep associated with a loss of conscious awareness. Dream reports obtained after awakenings from REM sleep are full of vivid sensory and emotional content (Hobson, 2002). In contrast, reports obtained after NREM sleep stages are of a more heterogeneous nature. Generally, the sleep stages defined by the AASM sleep staging criteria are associated with different behavioral and cognitive changes, as well as with different types of subjective conscious content reports obtained after sudden awakenings. Early sleep (N1 stage) is characterized by recollections of vivid hypnagogic hallucinations and lucid dreams (Domhoff, 2002; Kusse et al., 2011), cognitive operations similar to those performed during wakefulness (Stickgold et al., 2000; Wamsley et al., 2010) and in some cases a failure to recognize a prior state of sleep (Tononi, 2009). It is known that the cortex can remain in a state similar to wakefulness for minutes after the thalamic deactivation occurring during sleep onset (Magnin et al., 2010), which could be behind the conscious content present during early sleep. On the other hand, subjects awakening from deeper (N2/N3) sleep stages often do not report any conscious content prior to the awakening (Hobson, 2002). This difference likely underlies preserved frontoparietal connectivity during early sleep (Larson-Prior et al., 2009), as well as a functional network modularity close to that of wakefulness (Tagliazucchi et al., 2013).

In spite of a dramatically diminished conscious awareness, dream reports are not completely absent from the deepest sleep stages (Tononi, 2009). These reports are less lengthy than those obtained after REM sleep and in general less vivid and elaborated (Antrobus, 1983). However, this possibility makes it necessary to monitor conscious content whenever sleep is used as a brain state model for unconsciousness. Given the difficulty of awakening every subject to ask them for reports of their conscious contents, this fact is often overlooked in sleep neuroimaging studies which aim to address changes associated with the unconscious state.

**Figure 4:**
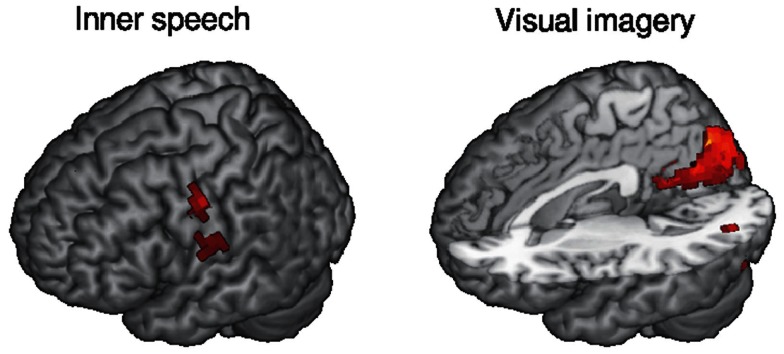
**Rendering of brain regions with mean BOLD activity (relative to wakefulness) during N2 sleep correlating with the reported level of inner speech/visual imagery experienced during the fMRI session (thresholded at *z* > 3)**. Subjective reports were collected retrospectively immediately after the fMRI session. fMRI data correspond to the same subjects reported in Tagliazucchi et al. (2013).

We asked our subjects to complete questionnaires after the EEG-fMRI scanning session, which include numerical ratings of conscious content of different nature, such as perceived visual imagery and inner speech. We then performed a correlation between these ratings and BOLD activity levels (relative to wakefulness) during N1, N2, and N3 sleep. A positive correlation with inner speech ratings was found near the Heschl’s gyrus in the temporal lobe and for visual imagery in the visual cortex (see Figure 4 for 3D renderings of the correlation maps). This result appears to confirm that conscious sensory content can be present during a sleep stage which is commonly associated with unconsciousness (N2 sleep).

This and other results do not suggest by any means that NREM sleep is an inadequate experimental model for unconsciousness. Caution is in order, however, and there is a need for experimenters to assess to what extent their subjects are truly unconscious.

## 6 Conclusion

In this review we have discussed the potential of NREM sleep neuroimaging to address fundamental predictions of theories about human consciousness. A relatively recent but constant methodological progress has allowed researchers to extract information from fMRI experiments going well beyond changes in BOLD signal amplitude. Many of these new methods are particularly useful to test the aforementioned predictions. For example, functional connectivity analyses reveal the disintegration of frontoparietal connectivity during deep sleep. Global functional connectivity networks studied using graph theoretical methods confirm that the deepest stages of NREM sleep are associated with increased functional modularity, in line with the predictions of the information integration theory. The recent discovery of a constantly changing BOLD connectivity could be fundamental to shed light on how different states of consciousness modulate the occurrence of transient synchronized states in the thalamocortical system.

We have emphasized that human consciousness is an emergent and global property of the human brain. The distributed nature of neural activity giving rise to consciousness is difficult to capture with neuroimaging methods which do not provide simultaneous coverage of the thalamocortical system with a reasonable spatial resolution. Currently, fMRI is one of the very few methods at our disposal which partially meets these requirements. However, the excitement about its possibilities should not prevent a discussion of its limitations. The most important is the limited temporal resolution (in the range of seconds), especially considering that neural processes giving rise to conscious awareness take place at the scale of hundreds of milliseconds. This leads to a careful interpretation of studies reporting functional connectivity changes, which need to be interpreted as an average over long periods of time. The number of samples required to obtain reliable connectivity estimates imposes a limit on the minimum temporal length during which functional connectivity can be resolved, a limit which is effectively longer than the actual temporal resolution of fMRI.

Finally, we closed our discussion with a note of caution regarding human sleep as an empirical model of absent consciousness. While human NREM sleep offers many advantages, it is also questionable to what extent it can be considered completely devoid of conscious content. Reports obtained after awakenings from early sleep confirm the presence of vivid sensory imagery. On the other hand, reports from deeper sleep stages are much shorter and can in fact be lacking any conscious content at all. We approached this problem by asking our subjects to fill a post-scan questionnaire which includes the rating of conscious sensory perception (visual and auditory) during the experiment. Correlations with BOLD activity were found in meaningful cortical regions during N2 (but not N3) sleep. This result suggests that conscious content can indeed be present during a typical NREM neuroimaging experiment. Thus, researchers should take this possibility into consideration when interpreting their results and, if possible, probe conscious content during the different sleep stages under study.

## Conflict of Interest Statement

The authors declare that the research was conducted in the absence of any commercial or financial relationships that could be construed as a potential conflict of interest.
